# Telerehabilitation for early-stage Parkinson's disease: A randomized controlled feasibility trial of individualised real-time physiotherapy delivered via a videoconference platform

**DOI:** 10.1177/1877718X261418551

**Published:** 2026-02-20

**Authors:** Rob Skelly, Fiona Lindop, Adam L Gordon, Neil H Chadborn, Manaal Malik, Kieron McFarlane, Lisa Brown, Jackie Beckhelling, Andrew Skeggs, Leanne Smith, Richard Walker

**Affiliations:** 1 Department of Medicine for Older People, University Hospitals of Derby and Burton, Derby, UK; 2 Specialist Rehabilitation, University Hospitals of Derby and Burton, Derby, UK; 3 Wolfson Institute of Population Health, Queen Mary University of London, London, UK; 4 Academic Centre for Healthy Ageing, Barts Health NHS Trust, London, UK; 5 NIHR Applied Research Collaboration East Midlands, Nottingham, UK; 6 School of Medicine, University of Nottingham, Institute of Mental Health, Triumph Road, Nottingham, UK; 7 Department of Neurology, University Hospitals of Derby and Burton, Derby, UK; 8 Department of Research and Development, University Hospitals of Derby and Burton, Derby, UK; 9 Department of Medicine, North Tyneside General Hospital, Rake Lane, North Shields, UK; 10 Population Health Sciences Institute, Newcastle University, Newcastle Upon Tyne, Tyne and Wear, UK

**Keywords:** Parkinson's, telerehabilitation, physiotherapy, fitbit, wearable, randomised controlled trial

## Abstract

**Introduction:**

Exercise can improve outcomes for people with Parkinson's. Telerehabilitation (TR) may lower costs and maximise clinician time but its efficacy for gait and balance in early Parkinson's is uncertain. We conducted a randomized controlled feasibility trial of individualised real-time physiotherapy delivered via videoconference.

**Methods:**

We recruited people with early (<4 years’ duration) Parkinson's from 2 English NHS hospitals. The TR group had 1 × 60 min, 4 × 30 min video calls and 2 × 10 min calls. These calls occurred within 12 weeks of randomization. Experienced physiotherapists prescribed individualized exercises. The usual care group received standard exercise advice from their physician. Physical activity was measured using Fitbit Inspire. A qualitative process evaluation was undertaken.

**Results:**

84 people were screened, 64 were eligible and 40 recruited. 21 and 19 were randomized to TR and usual care respectively. 90% of study instruments were completed per protocol.

Median [interquartile range; IQR] change in Unified Parkinson's Disease Rating Scale (UPDRS) at six months was −3.5 [−8 to 2.5] for the TR group and 7 [0 to 17] for usual care, effect size (Cohen's D = −0.537). Median [IQR] change in weekly step count was 4215 [−8769 to 19664] for the TR group versus −2185 [−10764 to 3143] for usual care (Cohen's D = 0.198). Participants found the intervention acceptable. Most participants were confident in using the videoconference systems.

**Conclusion:**

A definitive trial of TR for early Parkinson's is feasible. UPDRS and step count are suitable outcomes.

## Introduction

For people with Parkinson's, exercise can improve balance,^1^ reduce falls,^2^ improve sleep,^3^ reduce risk of depression and cognitive decline^4–6^ and may possibly slow disease progression.^7^ The role of physiotherapy in the treatment of falls and freezing of gait is well established^8,9^ but the role of early physiotherapy in the absence of recurrent falls and gait freezing is uncertain. A case for this can be made as people with early Parkinson's are less physically active than healthy controls and fewer than 50% reach recommended levels of physical activity.^10^ In addition gait changes occur early in Parkinson's^11^ so intervention at this stage may be beneficial. Furthermore, physiotherapist-supervised exercise may be more effective than self-supervised exercise.^12^

Telerehabilitation (TR) describes remote delivery of therapy services such as assessment, monitoring and training using telecommunications technology which may include use of telephone, video calls and online media.^13^ It may be individual or group, real time or use recordings, and be delivered with or without physiotherapist supervision. It may, or may not, involve use of other technologies such as virtual reality or wearable activity monitors.^14^ It may have some advantages over face-to-face therapy including reduced travel time for staff and patients, reduced infection risk and lower cost.^15,16^ Moreover, telerehabilitation is acceptable to people with Parkinson's and is feasible and safe.^17^ A recent review of telerehabilitation by videoconferencing for balance and gait concluded that efficacy was uncertain and found no studies using wearable technology to measure outcomes.^18^

We wanted to assess preliminary efficacy and acceptability of telerehabilitation for people with early Parkinson's (without recurrent falls or gait freezing and within 4 years of diagnosis). Specifically we wanted to investigate preliminary efficacy of an individualized physiotherapy intervention delivered by a videoconference platform to people with early Parkinson's in increasing physical activity levels and improving motor function and quality of life compared to usual care. Usual care was physician advice. A variety of outcomes measures were used and we aimed to identify potential primary outcome measures for a definitive trial. Here we report a randomized controlled feasibility trial with the primary objective of reporting feasibility outcomes for a future definitive trial. We also report a preliminary exploratory analysis of clinical outcomes at baseline (before the intervention) and at 3 and 6 months with estimation of effect size where appropriate. We also report outcomes of a qualitative process evaluation.

## Methods

*Setting.* The study took place at 2 centres in England: University Hospitals of Derby and Burton NHS Foundation Trust, and Northumbria Healthcare NHS Foundation Trust. Participants were recruited during the COVID-19 pandemic from Parkinson's Clinics run at the 2 centres between 5^th^ October 2021 and 8^th^ February 2024. Participants were recruited during periods of lockdown and periods with fewer restrictions on movement. The physiotherapy intervention was conducted using NHS Attend Anywhere or DrDoctor videoconferencing platforms. Attend Anywhere is a secure NHS video call service for patients with pre-arranged appointment times. The service is easy to use and highly acceptable to patients.^19^ Information about this service was available to participants at www.uhdb.nhs.uk/attend-anywhere. Likewise DrDoctor offers secure videoconferencing and information was available to participants at https://www.northumbria.nhs.uk/patients-and-visitors/patient-portal/video-consultations-drdoctor.

*Study population*. The study population were people with Parkinson's aged 18 years or more, diagnosed within the last 4 years according to UK Brain Bank criteria, Hoehn-Yahr stages 1–3, on stable Parkinson's medication (or not on Parkinson's medication) and independently mobile. Four years was used as a convenient cut-off time to define early Parkinson's and aligns typically with Hoehn-Yahr stages 1–2.^20^

### Study design

We conducted a feasibility trial of telerehabilitation physiotherapy for early Parkinson's versus usual care. This was a randomised controlled trial (RCT) with blinded assessment of outcomes and a parallel group design. Patients were followed up at 3 and 6 months after the baseline visit. We also conducted a qualitative process evaluation. This involved semi-structured interviews with a sample of participants in the intervention arm. We asked their views about acceptability, outcomes and study processes.

### Eligibility and recruitment

The recruitment target was 40 participants. We excluded subjects who lacked capacity to consent, who had more than 1 fall in the last 3 months, who had freezing of gait, or who were near the end of life as determined by criteria for commencement of Gold Standards Framework.^21^ We also excluded those who had already received physiotherapy for Parkinson's. Lack of a suitable device and lack of broadband were not exclusions as cellular tablet devices with adequate data allowance were available for loan. A member of the patients’ existing clinical team identified potential participants and gave them or sent them a participant information sheet. A member of the team contacted them after 1–2 weeks to see if they wished to participate in the study. We also publicised the study using posters and leaflets displayed in our outpatient clinics, and online via www.derbyparkinsons.com, and the funder's website, www.parkinsons.org.uk. Eligibility was checked by the research nurses who also took written informed consent. If a participant had a family caregiver, the caregiver also had the option of consenting to a caregiver assessment as part of the evaluation of the intervention. Participants were allocated on a 1:1 ratio (TR versus usual care), stratified by recruiting site using mixed blocks randomisation. The randomisations were generated using the randomisation module of DACIMA, a web-based platform for electronic data capture and clinical data management. The block sizes were 2 and 4.

The participants and the clinical staff administering the intervention were not blinded to the intervention but trial outcomes were assessed by blinded assessors. Participants were asked not to inform the research nurse of their treatment allocation. Inadvertent unblinding of the assessor by the participant was recorded. In the absence of an unblinding report, ongoing blinding of the assessor was assumed.

### Intervention

The intervention comprised an individualised video assessment by a physiotherapist lasting 60 min, 4 separate telerehabilitation treatment sessions lasting about 30 min and 2 video or telephone reviews lasting 10 min. The timing of these sessions was flexible and decided by the treating physiotherapist and the participants. Treatment sessions occurred no more frequently than weekly and usually treatment sessions were to be completed within 3 months of the baseline visit. All treatment sessions had to be completed before the 6-month follow up. The intervention was carried out by a physiotherapist experienced in Parkinson's which we defined as one seeing an average of 30 or more patients with Parkinson's per annum for at least 1 year. The initial video assessment included medical history, non-motor symptoms, gait, and posture, activities and exercise levels. Barriers to starting exercise and to continuing to exercise were considered, as was falls risk. Intervention was based on the core areas recommended by the European Physiotherapy Guideline for Parkinson's Disease: physical capacity, transfers, manual dexterity, balance and gait.^22^

All participants were taught a core set of exercises focusing on flexibility and amplitude of movement. Participants were given an individualized exercise prescription that also included strengthening exercises. Where possible, WHO recommendations for 150 min moderate, or 75 min vigorous, activity were included in the prescription. Prescriptions with regard to frequency and intensity of exercise were individualised, taking account of baseline activity levels and co-morbidities. Other potential interventions included: specific individualised exercises to improve muscle strength, endurance and posture; hand exercises practicing large movements; gait re-education, including cueing where indicated; balance training and falls prevention interventions; strategy training for transfer problems. While the focus of the intervention was physical therapy, education about Parkinson's, signposting to online resources such as Parkinson's UK and to local exercise groups, and informal psychological support was provided as needed. The physiotherapy lead for the project communicated with other therapists to explain the core elements of the intervention and encourage a consistent approach. We have used the TiDIER checklist in reporting this intervention (Appendix 1 online).^23^

### The comparison group

Participants in the standard care group received usual advice from their doctor/nurse about exercise and physical activity. They did not necessarily have physiotherapy but were referred for standard NHS physiotherapy if they developed a gait disturbance or recurrent falls. The treating clinician was allowed to refer for physiotherapy if they determined it was needed because of a change in the participant's condition.

### Outcomes measured

We collected the following feasibility outcomes: completeness of data collection for clinical outcomes; adherence of clinical staff to intervention protocol; length of waiting time from enrolment to delivery of intervention; change from baseline to follow up time points for all clinical outcomes, the standard deviation, and the effect size of clinical outcomes. Further feasibility outcomes included the number of screened subjects, the proportion of screened subjects who were eligible and the proportion of eligible subjects who consented to participate. We documented assessor unblinding.

Practicalities of using commercially available activity monitors as an outcome measure were documented. We used interviews to assess acceptability of the telerehabilitation intervention, suitability of personalized exercise prescriptions and barriers to engagement. Clinical outcomes were: Parkinson's Disease Questionnaire 39 (PDQ-39)^24,25^; Unified Parkinson's Disease Rating Scale (UPDRS)^26^; a modified version validated for remote administration was used - this version excludes the retropulsion test and assessment of tone^27^; activity as captured using 7-day activity diaries; step count and activity intensity (7-day) using Fitbit Inspire HR activity monitors which have been successfully used in Parkinson's research previously^28,29^; Short Form 12 (SF-12) measuring caregiver well-being, where a caregiver is available^30^; single leg stance test [SLST],^31^ a test of balance; five-times-sit-to-stand test FTSTST,^32^ another test of balance; and number of falls. Participants were asked to keep an activity diary for 7 days at baseline, 3 months and six months and record in the diary minutes of activity and perceived exertion using the Borg scale. Outcome assessments could be done via video call or in person at the discretion of the research site and participant. All outcomes were conducted in practically-defined “on” state: if they were on dopaminergic medication, they continued to take scheduled medication on the day of assessment and if they had on-off motor fluctuations they were assessed when medication was working well.

### Safety

In the intervention group falls risk was assessed at baseline by experienced physiotherapists. This included medical and medication history and clinical tests of balance. In the usual care group, participants could access physiotherapy outside of the study at the discretion of their usual clinician. Adverse events and serious adverse events were assessed for severity and causality and rigorously reported.

### Statistical plan

A sample size of 40 patients would enable a difference between baseline and 6 months follow-up in mean PDQ-39 of 8.3 to be detected with 80% power using a paired t-test with a 0.05 two-sided significance level. This was assuming a standard deviation of the difference between baseline and 6 months of 18, as reported by Ferrazzoli et al.^33^ However, as this was a feasibility study, the significance test was not going to be conducted (and has not been) but this was used as an indication of a suitable sample size.

Descriptive statistics are presented to summarise the distribution of baseline variables across each of the randomisation groups. Continuous variables are reported with medians and interquartile ranges (IQR) and categorical variables with frequencies and percentages. A Consolidated Standards of Reporting Trials (CONSORT) flow diagram was produced. Following international statistical guidelines,^34^ as this was a feasibility randomised controlled trial, hypothesis testing was not performed. The median and IQR change from baseline to follow up time points, the standard deviation, and the effect size of all clinical outcomes are presented per group. The effect sizes were calculated using Cohen's D ((M1-M1) / SD) where: M1 = mean of group 1, M2 = mean of group 2, SD = standard deviation. If the standard deviations were similar, the pooled SD was used, otherwise the calculation for unequal standard deviations was used.

Descriptive statistics are presented per group for the types of activity, the minutes of activity per week by the Borg perceived exertion scale using the following pre-set categories: Borg 6–12, light perceived exertion; Borg 13–14, moderate perceived exertion; Borg 15 −20, hard perceived exertion. The primary analysis of all outcomes was carried out on the intention to treat principle, retaining patients in their initially randomised group irrespective of any protocol violations. Pre-defined feasibility progression targets were: 25% for the proportion of screened individuals who were eligible to take part; 80% for the proportion of study instruments completed; and 75% for the proportion of participants completing the intervention and study assessments.

### Qualitative process evaluation

A sample of participants were interviewed for the process evaluation via Microsoft Teams or via telephone. Semi-structured interviews explored technical issues or problems and how participants found the digital remote consultation. Participants were asked for their views on advice or suggestions of the physiotherapist. Finally, participants were asked for any comments about the process of participation and whether they had any thoughts or suggestions on outcome measures. The interviews were recorded and transcribed for thematic analysis. Inductive thematic analysis was carried out by in-depth reading of transcripts and discussion within the team (MM, KM, AG, NC). The methodological framework was process evaluation; thus whilst we explored participants’ construction of meaning, this was within the constrained context of an experimental study.^35,36^ Broad topic areas for analysis followed themes of the interview schedule; digital confidence and physical activity. Emergent themes were also developed.

### Ethics

Ethical approval for this study was provided by West Midlands - South Birmingham Research Ethics Committee (Ref: 20/WM/0078).

## Results

1. Summary of participants recruited

**Figure 1.**
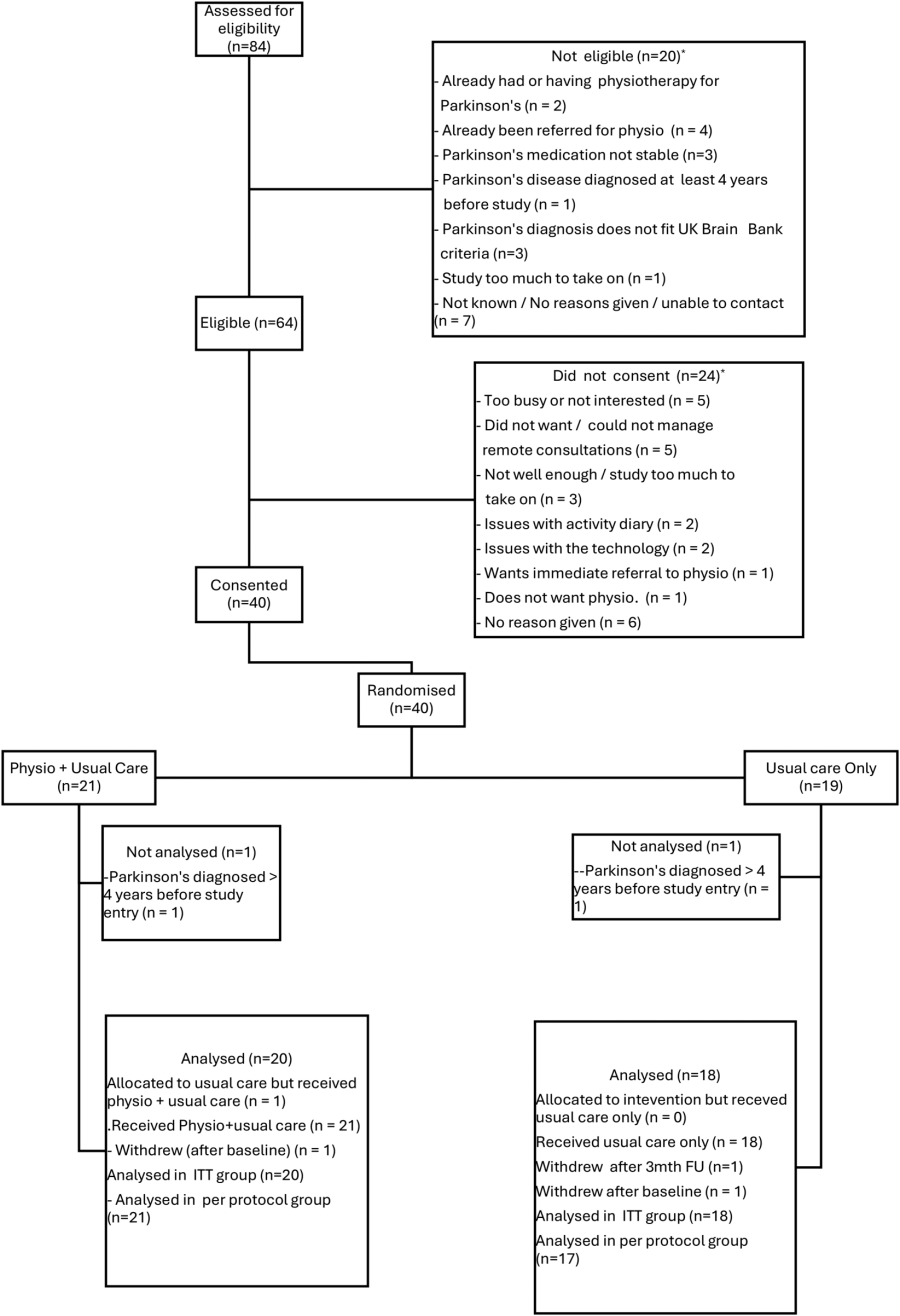
Consort diagram.

Eighty-four patients were assessed for eligibility and 64 were identified as eligible to participate. Of these, 40 consented and were randomized. Twenty-one participants were randomized to TR and 19 to usual care. Three patients withdrew, one from the TR group and 2 from the usual care group. When analysing the demographic data, two patients (1 usual care, 1 TR) were found to have been diagnosed with Parkinson's more than 4 years before they started the study and both were excluded from the analysis. (See Figure 1 Consort Diagram.)

2.Baseline Characteristics

Baseline characteristics of the TR and control groups are compared in Table 1. A statistical analysis was not done. The TR group and controls were not entirely similar with respect to baseline characteristics. Although age, gender distribution and years since diagnosis were all similar, there were differences in baseline median UPDRS and PDQ-39. At baseline for UPDRS score the TR group median (IQR) was 43 (22.5 to 66) versus 32 (26 to 48) in the usual care group. The higher UPDRS score indicates worse control of Parkinson's symptoms at baseline in the TR group. Conversely for PDQ-39, the TR group baseline median (IQR) was 6.9 (4.69 to 20.65) versus 11.25 (7.60 to 21.25) in the usual care group. Higher PDQ-39 scores equate to worse quality of life. Exploration of the subscores of the 2 scales showed that the UPDRS scores differed mainly in UPDRS motor examination subsection and the PDQ-39 scores differed mainly in the “stigma” subsection.

3.Feasibility outcomes with progression criteria

**Table 1.** Baseline characteristics of telerehabilitation and usual care groups.

	Telerehabilitation group n = 20	Usual care group n = 18
Age median (IQR)	68.0 (67.0 to 73.5)	71.5 (60 to 77.0)
Female Sex n (%)	6 (30)	4 (22)
Hoehn-Yahr Stage		
1 & 2	18 (90)	17 (94)
3	2 (10)	1 (6)
Time since diagnosis (years)		
< 1 year	14 (70)	13 (72)
>1 year	6 (30)	5 (28)
UPDRS	43 (22.5–66)	32 (26 to 48)
MoCA median (IQR)	27 (25.5 to 29)	27 (24 to 28)
PDQ-39	6.9 (4.69 to 20.65)	11.25 (7.60 to 21.25)
Co-morbidities	3 (1 to 4.5)	2 (1 to 3)
Taking L-dopa n (%)	18	12
Taking dopamine agonist n (%)	1	2
Taking rasagiline	0	3
L-dopa equivalent daily dosage (mg)	300 (175 to 325)	300 (100 to 300)

UPDRS = Unified Parkinson's Disease Rating Scale, possible score range 0–252; MoCA = Montreal Cognitive Assessment, possible score range 0–30; PDQ-39 = Parkinson's Disease Questionnaire 39, possible score range 0–100; L-dopa = levodopa.

Feasibility progression criteria were met: the proportion of screened individuals who were eligible to take part was 74%, the proportion of completed study instruments was 90%; and the proportion of participants completing the intervention and study assessments was 80%.

Of those in the TR group, all had 7 TR interventions but some had the 2 brief catch calls as telephone contacts rather than video calls as per an earlier version of the protocol. Two participants in the TR group and 1 in the usual care group had physiotherapy intervention outside of the study. Feasibility outcomes are shown in Table 2.

4.Qualitative Process evaluation

**Table 2.** Feasibility outcomes.

Screened individuals that were eligible	64/84 (74%)
Eligible individuals consented	40/64 (62.5%)
Participants completing all study interventions and assessments	16/20 (80%)
Proportion of completed study instruments	779/865 (90%)
Completion of caregiver assessments	108/114 (95%)
Unblinding of assessor	8/38 (21%)
Usable activity monitor data at baseline	28/38 (74%)
Usable activity monitor data at baseline and 3 months	18/38 (47%)
Usable activity monitor data at baseline and 6 months	20/38 (53%)
Median [IQR] time (minutes) to download baseline activity monitor data	10 (10–15)

Semi-structured interviews were conducted with 14 participants and transcripts were interpreted using thematic analysis. The a priori themes that we explored through our interview schedule were digital confidence and views on physical activity. Emergent themes were identified; coping with impairments of comorbidity and coming to terms with the diagnosis of Parkinson's. Although we were sensitive to digital divide for older people, our participants were mainly positive about the interaction, for example saying that it saved inconvenience of travelling into clinic; this may be indicative of ages of participants being younger due to being recently diagnosed. Some technical difficulties were experienced, but these were often addressed by the research team. A small number reported a slow internet connection which reduced quality of communication. Exploring the intervention, participants valued the interaction with the physiotherapists. At this early stage, it enabled participants to come to terms with the diagnosis and consider enhancing their physical activity to delay progression of symptoms. The personalized intervention enabled some participants to gain benefit for relieving specific motor symptoms, and others with co-morbidities benefited from learning ways to exercise that accommodated these impairments. Further, some participants valued personal recommendations of publicly available media demonstrating a particular exercise. In relation to outcomes, the participants were satisfied with most of the measures in the study. However there was one frustration with the activity monitor devices; because these had the display covered, the participants could not get feedback on their progress (the research team had decided to cover displays to avoid feedback influencing the intervention).

We also conducted one interview with one of the physiotherapists delivering the intervention (others were not available). One technical limitation to note was the limitation of range of view from, for example a laptop on a desk. This could be problematic for assessing range of movement and posture of the participant. A table of quotes from interviews is included as Appendix 2.

5.Clinical Outcomes

Median [IQR] change in UPDRS at six months was −3.5 [−8 to 2.5] for the TR group and 7 [0 to 17] for usual care, effect size (Cohen's D = −0.537). Median [IQR] change at 6 months in weekly step count was 4215 [−8769 to 19664] for the TR group versus −2185 [−10764 to 3143] for usual care (Cohen's D = 0.198). The proportion of participants reaching activity targets did not change for either group. Clinical outcomes are summarized in Table 3. There was no clinically meaningful difference in change in single leg stance time or FTSTS at 6 months. Activity diary data showed most activity was of light intensity throughout the study in both groups. The proportion of people doing at least some moderately vigorous activity at baseline was 83% in the TR group and 71% in the usual care group. This proportion did not increase form baseline in either group at 3 months or 6 months. In the TR group 4 (21%) participants experienced at least 1 fall versus 3 (19%) in usual care group.

**Table 3.** Clinical outcomes.

	Telerehabilitation Group			Usual Care Group			Effect size (Cohen's D with equal variances)	
	Baseline	3 month change from baseline	6 month change from baseline	Baseline	3 month change from baseline	6 month change from baseline	3 month follow up	6 month follow up
PDQ-39 Median (IQR)	6.90 (4.69 to 20.65)	1.15 (0 to 5.63)	1.30 (−1.61 to 2.60)	11.25 (7.60 to 21.25)	1.77 (−6.51 to 5.78)	3.90 (−2.81 to 8.49)	0.0672	−0.2527
UPDRS Median (IQR)	43 (22.5 to 66)	−5.5 (−9 to 0)	−3.5 (−8 to 2.5)	32 (26 to 48)	−2 (−7 to 9)	7 (0 to 17)	−0.147	−0.539
Single leg stance in seconds. Median (IQR)	10 (2 to 30)	0 (−2 to 2)	0 (−1 to 2)	12 (3 to 30)	−1 (−5 to 0)	0 (−7 to 0.5)	−0.3813	0.505
Five Times Sit to Stand in seconds Median (IQR)	13 (12 to 18)	−2.5 (−4 to −1)	−−2 (−3.5 to 0)	12 (9 to 16)	0 (−1 to 0)	−1 (−3 to −1)	−0.2801	0.4054
Activity monitor data								
Participants with data	14	8	10	14	10	10		
Weekly step count Median (IQR)	39939 (28293 to 53311)	−1139 (−7051 to 4635)	4215 (−8769 to 19664)	35999 (21849 to 49151)	5269 (0 to 26391)	−2185 (−10764 to 3143)	−0.7446	0.1984
Weekly Minutes fairly active Median (IQR)	81 (9 to 125)	40 (−2 to 94)	−2 (−75 to 41)	71 (39 to 226)	3.5 (−13 to 44)	−7.5 (−27 to 40)	0.7177	0.0871
Weekly Minutes very active Median (IQR)	90 (26–151)	1 (−103 to 57)	−12 (−54 to 49)	94 (25 to 214)	18 (1 to 108)	5.5 (−54 to 65)	−0.6903	0.0046
Number meeting activity target (%)	10 (71)	5 (63)	7 (70)	9 (64)	6 (60)	6 (60)		
Number not meeting activity target (%)	4 (29)	3 (38)	3 (30)	5 (36)	4 (40)	4 (40)		

PDQ-39 = Parkinson's Disease Questionnaire 39, possible score range 0–100; UPDRS = Unified Parkinson's Disease Rating Scale, possible score range 0–252.

Caregivers (n = 38) wellbeing, assessed by SF-12, did not change in the TR group and reduced minimally in the usual care group. The median (IQR) baseline SF-12 score was 38.5 (31.5 to 44) for the TR group and 38.5 (36 to 41) in the usual care group. Median (IQR) change from baseline at 6 month follow up was 0 (−2 to 3) and −1 (−6 to 2) respectively, effect size −0.585.

6.Safety outcomes

Six participants experienced safety events (1 in the TR group and 5 in the usual care group). A total of 10 events were experienced, 1 in the TR group and 9 in the usual care group. Three serious adverse events occurred in the usual care group but these were not related to treatment: 1 hip fracture, 1 stroke, and 1 admission following a road-traffic accident.

## Discussion

The key findings of this randomized controlled feasibility trial of telerehabilitation for early-stage Parkinson's using a video-conferencing platform to deliver real-time, individualized physiotherapy were that it is possible to recruit people with early Parkinson's to a telerehabilitation study and that most study assessments and interventions could be completed. A qualitative process evaluation using semi-structured interviews showed that participants valued the interaction with the physiotherapist and they benefitted from personalized recommendations that took account of their co-morbidities and activity levels. Participants found that remote delivery of the intervention was convenient, for example in avoiding travel. Whilst some participants did experience technical problems, these were addressed by the research team and did not inhibit participation in the study. Participants had recently been diagnosed with Parkinson's, and several mentioned aspects of coming to terms with the diagnosis. This may indicate that the personalized intervention was able to support an adaptation of sense of self to the condition and, in particular, address concerns about physical activity and maintaining fitness with Parkinson's. In this study the telerehabilitation group's UPDRS improved at 3 months and remained lower (better) than baseline at 6 months. Step count improved at 6 months only. Because this is a feasibility trial these results are not generalizable but they help inform a potential future study.

An important feature of this trial was delivery of individual person-centred therapy. Individual therapy may have some advantages over group therapy in that the type, frequency, intensity and duration of exercise can be tailored to the individual.^1^ In this regard it differs from a recently published trial of TR in Parkinson's which compared group teleneurorehabilitation with in-person therapy and, after 12 weeks of therapy, found improvements in motor function, non-motor symptoms, quality of life and balance in both groups.^37^

Our results are consistent with systematic reviews of telerehabilitation which show improvements in quality of life, satisfaction, and motor and non-motor symptoms.^38,39^ Our results also build on smaller studies showing safety, acceptability and efficacy of individualised telerehabilitation for Parkinson's.^40,41^

Although activity monitors have been used effectively in Parkinson's research,^28^ we believe this is one of the first studies of a telerehabilitation intervention for Parkinson's that also used activity monitors to assess activity.^18^

Others have shown that TR via a videoconference platform using pre-recorded content is acceptable to people with Parkinson's.^42^ We used semi-structured interviews that indicated individualized real-time TR is also acceptable. Furthermore we established this in a different cultural context.

Many rehabilitation studies in people with Parkinson's use more intensive treatment schedules^37,43^ but it is important to be cognisant of costs and investigate sustainable lower intensity schedules.

Strengths of this study are: it included more than one site which provides important information from two diverse contexts. It identified outcome measures that are likely to be appropriate for a definitive trial (step count, UPDRS), and had blinded outcome assessment and interviews with participants. Use of activity monitors was novel. Furthermore, unlike many published clinical studies of Parkinson's, the age profile of the study population is similar to the age profile of Parkinson's in the UK where the estimated median age of disease onset is 72.^44^ An option to borrow cellular devices with an adequate data allowance meant individuals lacking devices or broadband could be included.

An important limitation of this study relates to the fact that both recruiting centres were English teaching hospitals, and both of which were run by study co-authors. Therefore, there may be issues about the feasibility of study selection, randomisation, intervention and follow-up procedures in other hospitals which were district generals, outside of England and where Movement Disorder services are not directly run by the study team. Even allowing for the higher prevalence of Parkinson's in men, the proportion of women recruited to the study was lower than expected. This is a common issue in Parkinson's research and could affect generalizability of results. To address this a definitive trial would need to adopt sex-specific recruitment targets. It might also recruit through female-specific support groups and include women with Parkinson's in the project team to co-design a definitive study.^45^

It is important to bear in mind that this study was conducted during the COVID pandemic. Heightened concerns about infection risk from in-person consultations may have boosted interest in telerehabilitation. It is not clear if people with Parkinson's will be as motivated in times with lower infection risk.

The COVID pandemic also meant that it was not practical to choose in-person physiotherapy as the control group but this could potentially be considered for a future definitive trial.

Whilst participants found the intervention acceptable we note around a third of eligible people declined to take part. The reasons given (concerns about technology, poor digital literacy, lack of time) align with other studies.^41,46^ It seems likely some people with Parkinson's will not want telerehabilitation.

The amount of usable activity monitor data was disappointing. The trial management committee noted this issue early in the trial and revised the activity monitor guidance sheet given to participants. Per protocol, in order to avoid the chance of activity monitoring data actually influencing level of activity, we covered the face of the activity monitor. In retrospect, we surmise that covering the face meant the participant may not have known if the monitor was on and charged. As activity monitor data were incomplete a future trial would need a more reliable process for ensuring activity is recorded and downloaded.

## Conclusion

An assessor-blinded RCT of telerehabilitation for early Parkinson's would be feasible to conduct, and acceptable to participants. This study showed that a person-centred intervention could be delivered, and possibly the intensity of the intervention could be increased where individually appropriate. Subject to consultation with people with Parkinson's, appropriate outcome measures should include UPDRS and step count. Further consideration should be given to improving the process for activity monitor data acquisition; should participants be able to view the stepcount, where this could give feedback motivation to the participant, or should this remain ‘blinded’ as an objective outcome measure.

## Supplemental Material

sj-docx-1-pkn-10.1177_1877718X261418551 - Supplemental material for Telerehabilitation for early-stage Parkinson's disease: A randomized controlled feasibility trial of individualised real-time physiotherapy delivered via a videoconference platform Supplemental material, sj-docx-1-pkn-10.1177_1877718X261418551 for Telerehabilitation for early-stage Parkinson's disease: A randomized controlled feasibility trial of individualised real-time physiotherapy delivered via a videoconference platform by Rob Skelly, Fiona Lindop, Adam L Gordon, Neil H Chadborn, Manaal Malik, Kieron McFarlane, Lisa Brown, Jackie Beckhelling, Andrew Skeggs, Leanne Smith and Richard Walker in Journal of Parkinson's Disease

sj-docx-2-pkn-10.1177_1877718X261418551 - Supplemental material for Telerehabilitation for early-stage Parkinson's disease: A randomized controlled feasibility trial of individualised real-time physiotherapy delivered via a videoconference platform Supplemental material, sj-docx-2-pkn-10.1177_1877718X261418551 for Telerehabilitation for early-stage Parkinson's disease: A randomized controlled feasibility trial of individualised real-time physiotherapy delivered via a videoconference platform by Rob Skelly, Fiona Lindop, Adam L Gordon, Neil H Chadborn, Manaal Malik, Kieron McFarlane, Lisa Brown, Jackie Beckhelling, Andrew Skeggs, Leanne Smith and Richard Walker in Journal of Parkinson's Disease
