# Supplementary material for: Telerehabilitation for early-stage Parkinson's disease: A randomized controlled feasibility trial of individualised real-time physiotherapy delivered via a videoconference platform
Source: J Parkinsons Dis. 2026 Feb 20;16(3):578–88. doi: 10.1177/1877718X261418551 (PMC13347594; doi:10.1177/1877718X261418551)
Supplement: sj-docx-2-pkn-10.1177_1877718X261418551 - Supplemental material for Telerehabilitation for early-stage Parkinson's disease: A randomized controlled feasibility trial of individualised real-time physiotherapy delivered via a videoconference platform [file sj-docx-2-pkn-10.1177_1877718X261418551.docx]

Appendix 1 Quotes from semi-structured interviews (process evaluation)

Technical problems:

- My computer's rather slow anyway, so I don't know whether that was the problem. With [research nurse], we were freezing all the time. It just didn't feel right. Sometimes the quality wasn't very good.
- I mean it's probably my computer and the service isn't very good where I live anyway.

Digital confidence

- I've never been very good with computers…I think I've always had like some kind of mental block with computers and technology in general
- They gave me this tablet I'm looking forward to using [it], which I've never done before, which is with the video call. So that'll be all new to me…
- I often have to get help for things that I'm not sure about

Interaction with physiotherapist – online versus in-person

- …didn't know her, I never met her before. I felt that was awkward to first meet on a video chat. We should have had a face to-face first.
- People can see you without travelling for two hours. It's just as easy as a phone call

Digital resources

- I am not techy, and I've never went on YouTube at all, they said go on YouTube and there are loads of exercises for Parkinson's. I saw all the exercise for Parkinson's for balance and for your neck and your hands. I started them straight away
- I think in some ways that needs to be explained. Why are you giving people this Fitbit and what you're monitoring?

Reflections on diagnosis that emerged through physiotherapy

- I want to do as much as I can for my family whilst I still can
- Just to keep strength in my legs. So that I can carry on walking and sort of maybe counteract some of the issues Parkinson’s disease can cause... so hopefully not to end up in a wheelchair, but you never know
- I haven’t actually come out as yet. Only close family know and my boss at work
- …if you exercise and, you know, carry on doing what I’m doing, I can live, you know, hopefully help me live a long life. And it doesn't have to define me and all that jazz. So, that was from the physio on this study.

Motivations and barriers for physical activity

- I’m just keeping myself pretty active to just keep my mind on track. I’m retired now so I'm just doing this to make sure I'm mentally alert.
- I had a knee replacement last year. That's affected things a lot because it is still rather stiff. That's what stopped me swimming.... I don't really like walking out on my own... it seems to be in the back of my mind quite a lot. But I think as well is, it's falling and landing on my knee
